# Association Between the Sulfur Microbial Diet and Risk of Colorectal Cancer

**DOI:** 10.1001/jamanetworkopen.2021.34308

**Published:** 2021-11-12

**Authors:** Yiqing Wang, Long H. Nguyen, Raaj S. Mehta, Mingyang Song, Curtis Huttenhower, Andrew T. Chan

**Affiliations:** 1 Division of Gastroenterology, Massachusetts General Hospital and Harvard Medical School, Boston; 2 Clinical and Translational Epidemiology Unit, Massachusetts General Hospital and Harvard Medical School, Boston; 3 Department of Biostatistics, Harvard T.H. Chan School of Public Health, Boston, Massachusetts; 4 Department of Nutrition, Harvard T.H. Chan School of Public Health, Boston, Massachusetts; 5 Department of Epidemiology, Harvard T.H. Chan School of Public Health, Boston, Massachusetts; 6 Broad Institute of MIT and Harvard, Cambridge, Massachusetts; 7 Department of Immunology and Infectious Disease, Harvard T.H. Chan School of Public Health, Boston, Massachusetts; 8 Channing Division of Network Medicine, Department of Medicine, Brigham and Women’s Hospital and Harvard Medical School, Boston, Massachusetts

## Abstract

**Importance:**

Sulfur-metabolizing bacteria that reduce dietary sulfur to hydrogen sulfide have been associated with colorectal cancer (CRC). However, there are limited studies investigating the association between diet and sulfur-metabolizing bacteria in the development of CRC.

**Objective:**

To develop a dietary score that correlates with gut sulfur–metabolizing bacteria and to examine its association with CRC risk.

**Design, Setting, and Participants:**

This prospective cohort study included data from the Health Professionals Follow-up Study (1986-2014), Nurses’ Health Study (1984-2016), and Nurses’ Health Study II (1991-2017). Participants were US male health professionals and female registered nurses who were free of inflammatory bowel disease and cancer at baseline, with a subsample of participants who provided stool samples from 2012 to 2014. Statistical analysis was conducted from September 1, 2020, to June 1, 2021.

**Exposure:**

A dietary pattern, assessed by a food-frequency questionnaire, that most correlated with 43 sulfur-metabolizing bacteria identified through taxonomic and functional profiling of gut metagenome data.

**Main Outcomes and Measures:**

Incident CRC.

**Results:**

Among 214 797 participants comprising 46 550 men (mean [SD] age at baseline, 54.3 [9.7] years) and 168 247 women (mean [SD] age at baseline, 43.0 [9.2] years), 3217 incident cases of CRC (1.5%) were documented during 5 278 048 person-years of follow-up. The sulfur microbial diet, developed in a subsample of 307 men (mean [SD] age, 70.5 [4.3] years) and 212 women (mean [SD] age, 61.0 [3.8] years), was characterized by high intakes of low-calorie beverages, french fries, red meats, and processed meats and low intakes of fruits, yellow vegetables, whole grains, legumes, leafy vegetables, and cruciferous vegetables. After adjustment for other risk factors, greater adherence to the sulfur microbial diet was associated with an increased risk of CRC, with a hazard ratio (HR) of 1.27 (95% CI, 1.12-1.44) comparing the highest vs the lowest quintile of the diet score (linear trend of diet score quintiles; *P* < .001 for trend). When assessed by anatomical subsites, greater adherence to the sulfur microbial diet was positively associated with distal CRC (HR, 1.25; 95% CI, 1.05-1.50; *P* = .02 for trend) but not proximal colon cancer (HR, 1.13; 95% CI, 0.93-1.39; *P* = .19 for trend).

**Conclusions and Relevance:**

Adherence to the sulfur microbial diet was associated with an increased risk of CRC, suggesting a potential mediating role of sulfur-metabolizing bacteria in the associaton between diet and CRC. Further research is needed to confirm these findings and to determine the underlying mechanisms.

## Key Points

**Question:**

Is there an association between a dietary pattern that correlates with sulfur-metabolizing bacteria in the gut and colorectal cancer risk?

**Findings:**

In 3 large prospective cohort studies of US men and women, greater adherence to a sulfur microbial diet characterized by high intakes of low-calorie beverages and red meats and low intakes of fruits and vegetables was associated with increased risk of colorectal cancer, after controlling for various risk factors.

**Meaning:**

This study suggests that sulfur-metabolizing bacteria may mediate the association between diet and colorectal cancer risk and could potentially be targeted for risk mitigation.

## Teaser

This cohort study uses data from the Health Professionals Follow-up Study, Nurses’ Health Study, and Nurses’ Health Study II to develop a dietary score that correlates with gut sulfur–metabolizing bacteria and to examine its association with colorectal cancer risk.

## Introduction

Diet is an important modifiable risk factor for colorectal cancer (CRC), the third most common cancer worldwide and the second leading cause of cancer death according to GLOBOCAN 2020.^1^ However, the roles of specific gut microbial activities in the diet-CRC association have not been thoroughly investigated. These gut microbial activities include the sulfur-metabolizing microbiota known to metabolize dietary sulfur to genotoxic hydrogen sulfide,^2^ which may promote inflammation, cause epithelial DNA damage, and increase CRC risk.^3,4^ In humans, protein has been shown to be associated with intestinal sulfide production,^2^ and an animal-based diet with high protein and high fat contents has been shown to enrich gut microbiome–encoding sulfite reductases.^5^

A recent study developed a de novo dietary score associated with the enrichment of sulfur-metabolizing gut bacteria using data from the Men’s Lifestyle Validation Study (MLVS) and found that this sulfur microbial diet score, characterized by high intakes of processed meats and low-calorie beverages, was associated with increased risk of distal CRC in men.^6^ However, because there are potential sex-based differences in nutrient metabolism^7^ and CRC risk,^8^ a study leveraging a more diverse population is needed to confirm these findings. Cohort studies^9,10^ and reviews^8,11^ have reported that, compared with men, women have a lower overall incidence of CRC but a higher risk of proximal colon cancer, which tends to be more advanced and less differentiated than distal colon cancer.^8,10,12^ In addition, there are potential sex differences in the gut microbiome in response to diet.^13,14^ For example, a study using an interleukin 10–deficient murine colitis model found that, in male mice, high-dose dietary fiber supplementation was associated with increased microbial alpha diversity and reduced colonic interleukin 12p70, while in female mice, there was no change in alpha diversity, microbial relative abundance, or colonic inflammation markers.^13^

Therefore, we expanded this previous sulfur microbial diet study in men^6^ by combining the MLVS and the Mind Body Study (MBS) of female registered nurses^15^ to develop an updated sulfur microbial diet score using an analytical approach that accommodates multiple cohorts and sparse microbiome data.^16^ We then examined the association of this updated sulfur microbial diet score with CRC risk in 3 large prospective cohorts of US men and women.

## Methods

### Study Population

We used data from the Health Professionals Follow-up Study (HPFS), the Nurses’ Health Study (NHS), and the Nurses’ Health Study II (NHSII). The HPFS recruited 51 529 male health professionals aged 40 to 75 years at enrollment in 1986. The NHS enrolled 121 700 female registered nurses aged 30 to 55 years when initiated in 1976. The NHSII began in 1989 and consisted of 116 429 female registered nurses aged 25 to 42 years. Response rates were more than 90% in all cohorts.^17,18,19^ In each cohort, detailed questionnaires on demographics, lifestyle risk factors, and disease information were administered every 2 years, while dietary intake was assessed every 4 years by a validated semiquantitative food frequency questionnaire (FFQ).^20^ We included participants who answered the baseline FFQ in the 1986 HPFS, the 1984 NHS, and the 1991 NHSII. Participants who had ever received a diagnosis of CRC, inflammatory bowel disease, or other cancer except for nonmetastatic skin cancer; only returned baseline questionnaires; or had missing date of birth were excluded at baseline. These studies were approved by the institutional review boards of the Brigham and Women’s Hospital and Harvard T.H. Chan School of Public Health, and those of participating registries as required. Informed consent was implied by return of study questionnaires. This report followed the Strengthening the Reporting of Observational Studies in Epidemiology (STROBE) reporting guideline for cohort studies.

From 2012 to 2013, longitudinal stool samples were collected from men in the MLVS nested within the HPFS, and from 2013 to 2014 longitudinal stool samples were collected from women in the MBS nested within the NHSII. Study design and biospecimen collection of these 2 studies have been described previously.^6,15^

### Stool Sample Collection and Processing

Men’s Lifestyle Validation Study and MBS participants were requested to provide 2 stool specimens from consecutive bowel movements at home 1 to 3 days apart following a collection protocol that has been validated against fresh-frozen sample collections.^21,22,23^ A second pair of samples was collected approximately 6 months later following the same instructions. Participants were also asked to complete questionnaires detailing the collection time, stool appearance, and lifestyles.^6,15^ Samples were shipped overnight to the laboratory the next day and stored at −80 °C until sequencing at the Broad Institute.^15^ As described previously,^24^ we used the HiSeq paired-end shotgun sequencing platform (Illumina Inc) to generate metagenomes and the bioBakery2 metagenome workflow^25^ to yield the taxonomic profile. We subsequently removed 5 samples from MBS participants with implausible microbial composition (eg, >99% Firmicutes) or low passing filter reads (<100 000) and 1 sample from an MLVS participant because of a prior history of total colectomy.

### Development of the Sulfur Microbial Diet Score

A prior study has identified 43 sulfur-metabolizing bacteria (eTable 1 in the Supplement) based on pathway search and comprehensive literature review.^6^ We log_10_-transformed the mean relative abundance of these bacteria across repeated samples to minimize intraindividual variability. After each round of stool collection period, MLVS and MBS participants completed a semiquantitative FFQ using standard portion sizes (eg, 2 slices of bacon), indicating their frequency of consumption for each food item in 9 options ranging from “never or less than 1 time per month” to “6 times per day or more” during the past year. Consistent with prior methods,^26^ we converted intakes into servings per day, categorized foods into 33 predefined food groups, and averaged the intakes across both FFQs. In this developmental cohort, we conducted sparse canonical correlation analysis to select and obtain canonical weights for food groups and bacteria whose linear combinations maximized their correlations.^16^ Model parameters were optimized using 25 permutation tests. This updated method allows multiple cohorts to be assessed jointly, rather than singly.^16^ The performance of this method in high-dimensional microbiome data has been demonstrated in several studies.^27,28,29^ Pairwise association between each selected food group and bacteria was assessed using Spearman correlation analysis, with multiple testing corrected using the Benjamini-Hochberg false discovery rate.

### Assessment of Long-term Adherence to the Sulfur Microbial Diet

In the larger pooled cohort of HPFS, NHS, and NHSII participants (ie, testing cohort), we calculated the sulfur microbial diet score for each participant using the weighted sum of standardized intakes of selected food groups (in each cohort: mean = 0 and SD = 1). A higher score indicated a better adherence to the sulfur microbial diet, which represents a data-driven association with the relative abundance of sulfur-metabolizing bacteria in the gut. To capture long-term usual intake, we cumulatively averaged the sulfur microbial diet score across preceding FFQs updated at each questionnaire cycle and categorized the score into quintiles.

### Ascertainment of CRC

We ascertained incident CRC based on self-reported CRC cases from biennial questionnaires, medical records, pathology reports, reporting from next of kin, postal authorities, tumor registries, death certificates, and the National Death Index. Written permissions to obtain medical records or pathology reports were requested from participants who reported CRC diagnoses or the next of kin for lethal CRC cases. Study physicians who were blinded to participants’ risk factors reviewed relevant records to confirm cases and anatomical locations. We used CRC as the primary outcome and 2 anatomical subsites, proximal colon cancer and distal CRC, as secondary outcomes.

### Assessment of Covariates

We acquired self-reported CRC risk factors in biennial questionnaires, including age, family history of CRC, smoking status, smoking (in pack-years), physical activity (metabolic equivalent of task [MET] per week), and body mass index (BMI; calculated as weight in kilograms divided by height in meters squared). Self-reported race and ethnicity information was also collected to examine the association of race and ethnicity with lifestyle factors and risk of chronic disease. Because participants were predominantly White, we categorized them as White and all other race and ethnicity groups (American Indian/Native American, Asian, Black, Hawaiian, multiracial, and other). To capture overall dietary quality, we derived a Western dietary pattern score using principal component analysis, as previously described.^30^ We calculated mean pack-years of smoking, physical activity, BMI, total energy intake (kilocalories per day), and Western dietary pattern score across questionnaire cycles using cumulative mean method to minimize interindividual variation.

### Statistical Analysis

Statistical analysis was conducted from September 1, 2020, to June 1, 2021. We calculated person-time for each participant from study baseline until CRC diagnosis, death, or the end of follow-up (HPFS: January 31, 2014; NHS: June 30, 2016; and NHSII: June 30, 2017), whichever occurred first. We used age-adjusted and multivariable-adjusted Cox proportional hazards regression models to estimate hazard ratios (HRs) and 95% CIs of CRC associated with each quintile of the sulfur microbial diet score vs the lowest quintile. Linear trend was tested by modeling the median value of each diet score quintile as a continuous variable. The absence of effect modification by age as a proxy for calendar time (*P* = .16 for interaction in likelihood ratio test) indicated that there was no violation of the proportional hazards assumption. All models were stratified by age, questionnaire cycle, and cohort. In the multivariable model, the following potential confounders were selected a priori as covariates: race (White or other), BMI (continuous), family history of CRC (yes or no), prior endoscopy (yes or no), previous physical examination (yes or no), smoking status (never, past, or current), smoking pack-years (continuous), physical activity (continuous, METs per week), regular aspirin use (yes or no), regular nonsteroidal anti-inflammatory drug use (yes or no), menopausal hormone therapy (women only: premenopausal, never, past, or current), and total energy intake (continuous). For missing data, we carried forward nonmissing values from previous questionnaires and imputed with median values (0.002%-5% of all observations). We compared the current sulfur microbial diet score with the previous sulfur microbial diet score derived from the MLVS only^6^ and the Western dietary pattern score using Spearman correlation analysis.

We also analyzed the association between the sulfur microbial diet and CRC risk by anatomical subsites and sex, with heterogeneity assessed with the Cochran *Q* test. In addition, we investigated potential effect measure modification by age (≥60 vs <60 years), BMI (≥25 vs <25), regular aspirin use (yes vs no), smoking status (ever vs never), and family history of CRC (yes vs no), by comparing multivariable models with and without the interaction term of the sulfur microbial diet score and each stratification variable separately, using the likelihood ratio test. In sensitivity analysis, we additionally adjusted for the Western dietary pattern score in the multivariable model.

Analyses were performed using R, version 4.0.3 (R Group for Statistical Computing) and SAS, version 9.4 (SAS Institute Inc). Statistical tests were 2-sided with *P* < .05 considered significant.

## Results

**Figure 1.**
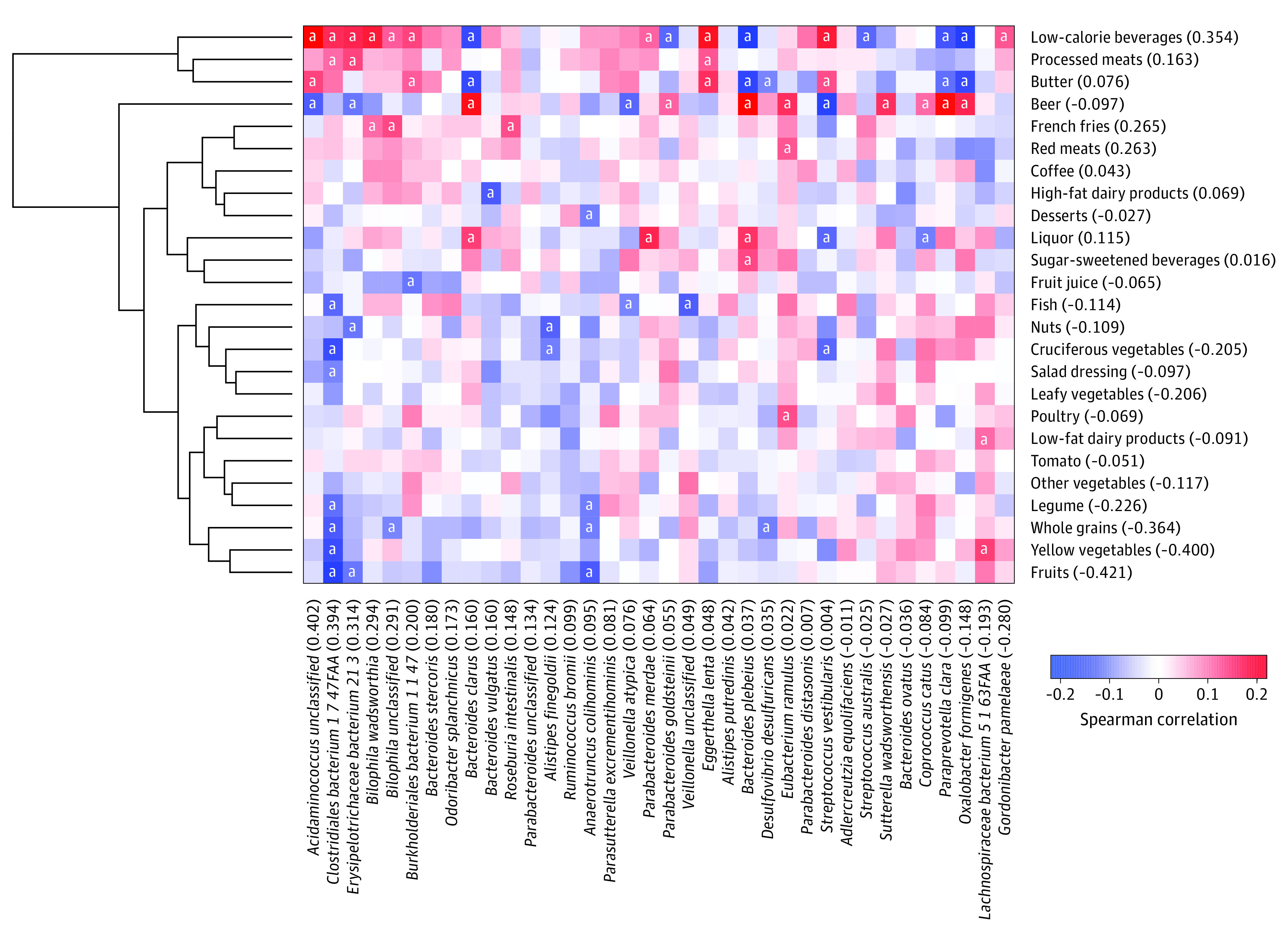
Sparse Canonical Correlation Analysis of Dietary Intake and Sulfur-Metabolizing Bacteria Pairwise Spearman correlations between selected food groups and bacteria. Numbers in parentheses are the weights for food groups and bacteria, whose linear combination maximizes the correlation (0.375). The food group weights were used to create the sulfur microbial diet score. ^a^Statistically significant correlations (Benjamini-Hochberg false discovery rate <0.25).

As is representative of the testing cohort, the 307 men (mean [SD] age, 70.5 [4.3] years) and 212 women (mean [SD] age, 61.0 [3.8] years) in the developmental cohort were predominantly White (290 of 307 [94.5%] to 204 of 213 [95.8%]), with a mean (SD) BMI of 25.5 (3.7) for men and 25.6 (5.3) for women at time of first sampling (eTable 2 in the Supplement). A total of 25 food groups and 36 sulfur-metabolizing bacteria were retained by sparse canonical correlation analysis, with an overall correlation of 0.375 (Figure 1). Representative food items for each food group are shown in eTable 3 in the Supplement. Food groups with positive weights, including low-calorie beverages, french fries, red meats, and processed meats, tended to be positively correlated with most of the sulfur-metabolizing bacteria, whereas food groups with negative weights, including fruits, yellow vegetables, whole grains, legumes, leafy vegetables, and cruciferous vegetables, tended to be negatively associated with most of the sulfur-metabolizing bacteria.

At baseline in the testing cohort of 46 550 men (mean [SD] age at baseline, 54.3 [9.7] years) and 168 247 women (mean [SD] age at baseline, 43.0 [9.2] years) (eFigure in the Supplement shows the sample flow), the resulting sulfur microbial diet score based on the weighted sum of standardized consumption of these food groups was modestly correlated with a predefined Western dietary pattern (Spearman correlation ρ = 0.26; *P* < .001) and the previous sulfur microbial diet score derived in men only (ρ = 0.61; *P* < .001).^6^ Men and women with greater adherence to the sulfur microbial diet tended to be younger, be less likely to have undergone endoscopy and physical examination, have higher BMIs, and have lower amounts of physical activity (Table 1; eTable 4 in the Supplement).

**Table 1.** Age-Adjusted Baseline Characteristics of the HPFS, NHS, and NHSII by Quintiles of Sulfur Microbial Diet Score

Characteristic	Sulfur microbial diet score, No. (%)				
	Quintile 1 (lowest)	Quintile 2	Quintile 3	Quintile 4	Quintile 5 (highest)
Men (HPFS)					
No.	9255	9415	9349	9287	9244
Age, mean (SD), y^a^	57 (9.8)	55.8 (9.7)	54.5 (9.6)	53.4 (9.4)	51 (8.9)
Race					
White	8279 (89.5)	8491 (90.2)	8417 (90.0)	8350 (89.9)	8380 (90.7)
Other^b^	976 (10.5)	924 (9.8)	932 (10.0)	937 (10.1)	864 (9.3)
BMI, mean (SD)	24.8 (3.3)	25.3 (3.1)	25.5 (3.2)	25.7 (3.3)	26.1 (3.3)
Physical activity, mean (SD), MET h/wk	28.6 (29.9)	22.8 (25.5)	19.7 (23.4)	17.3 (21.3)	15.3 (20.7)
Smoking status^c^					
Never	4869 (52.6)	4571 (48.6)	4095 (44.8)	3855 (41.6)	3372 (36.5)
Past	3641 (39.3)	3901 (41.4)	4028 (43.1)	3913 (42.1)	3931 (42.5)
Current	375 (4.1)	569 (6.0)	789 (8.4)	1143 (12.3)	1600 (17.3)
Pack-years among ever smokers, mean (SD)	14.7 (13.7)	15.8 (14.4)	16.8 (14.8)	17.9 (15.3)	19.4 (15.7)
Regular aspirin use	2687 (29.0)	2768 (29.4)	2742 (29.3)	2700 (29.1)	2714 (29.4)
Regular NSAID use	761 (8.2)	895 (9.5)	1003 (10.7)	1037 (11.2)	1072 (11.6)
Family history of CRC	1442 (15.6)	1420 (15.1)	1372 (14.7)	1377 (14.8)	1283 (13.9)
Prior endoscopy	2726 (29.5)	2683 (28.5)	2460 (26.3)	2226 (24.0)	2104 (22.8)
Prior physical examination	5712 (61.7)	5821 (61.8)	5674 (60.7)	5437 (58.5)	5176 (56.0)
Total calorie intake, mean (SD), kcal	2250.7 (624.7)	2002.5 (589)	1896.6 (576.8)	1843.5 (591.1)	1947.2 (640.9)
Western diet pattern score, mean (SD)^d^	−0.4 (0.8)	−0.2 (0.8)	−0.1 (0.8)	0.1 (0.9)	0.5 (1.0)
Women (NHS and NHSII)					
No.	33 585	33 585	33 708	33 681	33 688
Age, mean (SD), y^a^	44.3 (9.8)	43.6 (9.5)	43.1 (9.2)	42.5 (8.9)	41.6 (8.4)
Race					
White	31 941 (95.1)	32 127 (95.7)	32 225 (95.6)	32 225 (95.8)	32 276 (95.8)
Other^b^	1644 (4.9)	1458 (4.3)	1483 (4.4)	1456 (4.3)	1412 (4.2)
BMI, mean (SD)	24.1 (4.5)	24.2 (4.5)	24.4 (4.6)	24.6 (4.9)	25.2 (5.4)
Physical activity, mean (SD), MET h/wk	26.6 (31.8)	20.3 (24.1)	17.8 (22.2)	15.6 (20.9)	13.4 (18.9)
Smoking status^c^					
Never	19 892 (59.2)	19 578 (58.3)	19 200 (57.0)	18 293 (54.3)	16 683 (49.6)
Past	10 129 (30.2)	9574 (28.5)	8964 (26.6)	8339 (24.8)	7589 (22.5)
Current	3502 (10.4)	4359 (13.0)	5507 (16.3)	7011 (20.8)	9371 (27.8)
Pack-years among ever smokers, mean (SD)	21.8 (17.1)	23.8 (18.3)	24.4 (18.9)	26.1 (19.1)	28.0 (19.8)
Regular aspirin use	11 552 (34.4)	12 415 (37.0)	12 996 (38.6)	13 466 (40.0)	13 787 (40.9)
Regular NSAID use	7480 (22.3)	7987 (23.8)	8323 (24.7)	8450 (25.1)	9195 (27.3)
Family history of CRC	4319 (12.9)	4220 (12.6)	4257 (12.6)	4237 (12.6)	4016 (11.9)
Prior endoscopy	2330 (6.9)	2112 (6.3)	2025 (6.0)	1949 (5.8)	1762 (5.2)
Prior physical examination	28 467 (84.8)	27 899 (83.1)	27 628 (82.0)	27 028 (80.2)	25 915 (76.9)
Total calorie intake, mean (SD), kcal	2049.1 (527.6)	1816.9 (497.6)	1704.9 (504.4)	1616.2 (510.3)	1666.2 (555.7)
Western diet pattern score, mean (SD)^d^	−0.3 (0.9)	−0.2 (0.9)	−0.1 (0.9)	0 (0.9)	0.5 (1.1)
Menopausal hormone therapy					
Premenopausal	23 971 (71.4)	24 112 (71.9)	24 099 (71.4)	24 077 (71.5)	24 236 (72.0)
Never used hormones	4910 (14.6)	4881 (14.5)	5142 (15.3)	5269 (15.6)	5294 (15.7)
Past hormone user	1971 (5.9)	1957 (5.8)	1977 (5.9)	1907 (5.7)	1969 (5.8)
Current hormone user	2733 (8.1)	2635 (7.8)	2490 (7.4)	2428 (7.2)	2189 (6.5)

Abbreviations: BMI, body mass index (calculated as weight in kilograms divided by height in meters squared); CRC, colorectal cancer; HPFS, Health Professionals Follow-up Study; MET, metabolic equivalent task; NHS, Nurses’ Health Study; NHSII, Nurses’ Health Study II; NSAID, nonsteroidal anti-inflammatory drug.

^a^ Not adjusted for age.

^b^ Includes American Indian/Native American, Asian, Black, Hawaiian, and multiracial.

^c^ Percentages do not add up to 100% owing to missing data (3.8%-4.0% in men and 0.1%-0.2% in women), which were categorized into a missing category.

^d^ The predefined Western dietary pattern score was derived from principal component analysis.

We documented 3217 incident CRC cases (1.5%) among 214 797 total participants during 5 278 048 person-years and a median follow-up time of 26 years (IQR, 23-28 years) (Table 2). Greater adherence to the sulfur microbial diet was associated with increased risk of CRC, with an HR of 1.27 (95% CI, 1.12-1.44) (linear trend of diet score quintiles; *P* < .001 for trend) comparing the highest vs the lowest quintile of the diet score, after adjusting for a wide range of risk factors, including age, BMI, family history of CRC, physical activity, total energy intake, and smoking. When assessed by anatomical subsites, greater adherence to the sulfur microbial diet was associated with increased risk of distal CRC (HR, 1.25; 95% CI, 1.05-1.50; *P* = .02 for trend), but not proximal colon cancer (HR, 1.13; 95% CI, 0.93-1.39; *P* = .19 for trend).

**Table 2.** Hazard Ratios and 95% CIs of Incident Colorectal Cancer in the Pooled Analytic Cohort, by Quintiles of Sulfur Microbial Diet Score

Model	Sulfur microbial diet score, HR (95% CI)						*P* value for trend^a^	
	Quintile 1 (lowest)	Quintile 2	Quintile 3	Quintile 4	Quintile 5 (highest)		
Colorectal cancer							
Cases	650	693	642	636	596	NA	
Person-years	1 083 910	1 091 687	1 072 512	1 044 147	985 792	NA	
Adjusted for age^b^	1 [Reference]	1.12 (1.01-1.25)	1.14 (1.02-1.27)	1.24 (1.11-1.39)	1.42 (1.27-1.59)	<.001	
Multivariable adjusted^c^	1 [Reference]	1.10 (0.99-1.23)	1.10 (0.98-1.23)	1.16 (1.04-1.31)	1.27 (1.12-1.44)	<.001	
Proximal colon cancer							
Cases	264	265	238	232	201	NA	
Adjusted for age^b^	1 [Reference]	1.06 (0.89-1.26)	1.06 (0.88-1.26)	1.15 (0.96-1.37)	1.24 (1.03-1.50)	.02	
Multivariable adjusted^c^	1 [Reference]	1.05 (0.88-1.25)	1.03 (0.86-1.24)	1.10 (0.91-1.33)	1.13 (0.93-1.39)	.19	
Distal colon and rectal cancer							
Cases	297	319	305	298	281	NA	
Adjusted for age^b^	1 [Reference]	1.13 (0.96-1.32)	1.17 (1.00-1.37)	1.25 (1.06-1.47)	1.40 (1.19-1.66)	<.001	
Multivariable adjusted^c^	1 [Reference]	1.10 (0.93-1.29)	1.12 (0.95-1.32)	1.16 (0.98-1.38)	1.25 (1.05-1.50)	.02	

Abbreviations: HR, hazard ratio; NA, not applicable.

^a^ Trend test was performed using median value of each diet score quintile as a continuous variable.

^b^ Models were stratified by age, questionnaire cycle, and cohort.

^c^ Models were stratified by age, questionnaire cycle, and cohort, and adjusted for the following covariates: race, body mass index, family history of colorectal cancer, physical activity, smoking status, smoking pack-years, menopausal hormone use (women only), aspirin use, nonsteroidal anti-inflammatory drug use, prior endoscopy, recent physical examination, and total calorie intake.

**Figure 2.**
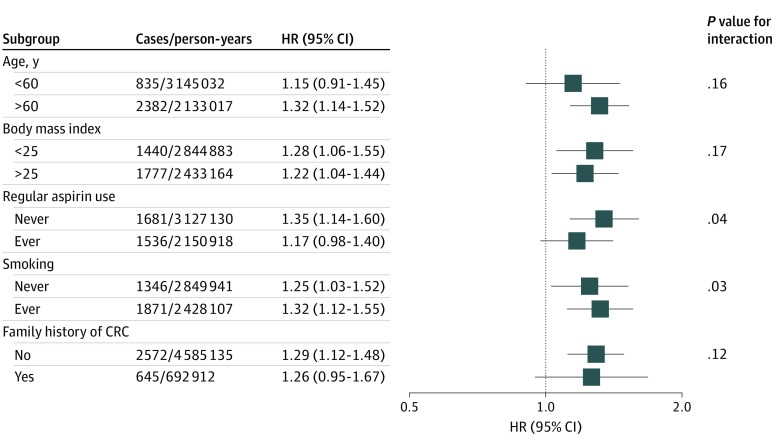
Stratified Analysis of the Association Between Sulfur Microbial Diet and Risk of Colorectal Cancer (CRC) Multivariable-adjusted hazard ratios (HRs) and 95% CIs comparing the highest quintile vs the lowest quintile of sulfur microbial diet score were calculated using Cox proportional hazards regression models stratified by age, questionnaire cycle, and cohort and adjusted for the following covariates: race, body mass index (calculated as weight in kilograms divided by height in meters squared), family history of CRC, physical activity, smoking status, smoking pack-years, menopausal hormone use (women only), aspirin use, nonsteroidal anti-inflammatory drug use, prior endoscopy, recent physical examination, and total calorie intake. *P* value for interaction was estimated using the likelihood ratio test comparing the model with and without the interaction term of the sulfur microbial diet score and the respective stratification variable.

Results for the overall CRC risk were similar between men and women (eTable 5 in the Supplement). After additional adjustment for Western dietary pattern score (eTable 6 in the Supplement), the positive association between sulfur microbial diet score and CRC risk remained but was slightly attenuated (HR, 1.21; 95% CI, 1.05-1.40; *P* = .01 for trend). In stratified analysis (Figure 2), the association between sulfur microbial diet score and CRC risk was slightly stronger among participants who did not regularly use aspirin and who ever smoked, compared with their referent counterparts.

## Discussion

In 3 large prospective cohorts of male and female health professionals, we found that long-term adherence to a sulfur microbial diet, which correlated with the relative abundance of sulfur-metabolizing gut bacteria, was associated with an increased risk of CRC. Compared with a previous study conducted in men,^6^ the current study used larger and more diverse data, used an analytical approach that accommodates multiple cohorts,^16^ and produced consistent results. Taken together, our results provide further evidence that sulfur-metabolizing bacteria may play a role in the diet-CRC association.

Studies have shown that an animal-based diet could enrich the gut microbiome–encoding sulfite reductases and sulfite-reducing bacteria,^5,31^ which generate hydrogen sulfide in sulfur metabolism, a harmful by-product that may induce DNA damage, disrupt the mucus bilayer, and promote inflammation and CRC.^3,4,32^ Similarly, our sulfur microbial diet score captured the positive correlations of the relative abundance of sulfur-metabolizing bacteria with red meats and processed meats, which are rich in both sulfur-containing amino acids and inorganic sulfur from preservatives. In addition, a diet that provides high levels of sulfur-containing amino acids contains high amounts of animal protein and fat and low amounts of fiber,^31^ which resembles a Western dietary pattern that has been implicated in the development of CRC.^33^ A recent study has demonstrated that a Western diet was associated with increased CRC risk in mice by reducing the expression of bile acid transporters,^34^ indicating a role of bile acids in the dietary cause of CRC. Although our sulfur microbial diet score was somewhat correlated with a predefined Western dietary pattern score,^26^ its associations with CRC risk were partially independent of the Western dietary pattern score, suggesting an interplay of multiple related mechanisms. One potential distinction between these 2 dietary pattern scores is the much higher consumption of low-calorie beverages (eg, low-energy cola) than sugar-sweetened beverages (eg, cola with sugar or fruit drinks) in the sulfur microbial diet, while the consumption of low-calorie beverages tended to be lower than consumption of sugar-sweetened beverages in the predefined Western dietary pattern.^26^ Prospective cohort studies have previously reported that low-calorie beverage intake was inversely associated with CRC-specific and all-cause mortality among patients with stage I to III CRC,^35^ as well as cancer recurrence and mortality among patients with stage III colon cancer.^36^ The mechanisms by which low-calorie beverage intake may modulate sulfur-metabolizing bacteria and its association with incident CRC risk rather than survival warrant further investigation.

In contrast, intake of cruciferous vegetables (eg, cabbage, broccoli, and kale), which are rich in the sulfur-containing glucosinolates and have been linked to reduced CRC risk,^37^ was negatively correlated with sulfur-metabolizing bacteria. Glucosinolates can be hydrolyzed to isothiocyanates by myrosinase-expressing gut microbiota.^38^ The anticarcinogenic effects of isothiocyanates and their downstream products have been demonstrated in several studies.^39,40,41^ Therefore, consistent with the prior study of the sulfur microbial diet,^6^ our results indicate that the dietary source of sulfur and the specific sulfur-containing compounds, instead of the total sulfur content, may determine the relative abundance of sulfur-metabolizing bacteria.

### Strengths and Limitations

Our study has some strengths, including large prospective cohorts with more than 25 years of follow-up, high follow-up rates, and regularly updated rich information on diet and health outcomes, limiting selection and recall bias. The inclusion of a well-characterized subcohort with repeated diet assessments and stool collections allowed us to specifically investigate the association between local microbial and dietary patterns and broader CRC risk. In addition, our concurrent assessment of lifestyle factors allowed us to control for a wide range of potential confounders, which did not materially alter our estimates comparing the multivariable-adjusted and age-adjusted models. These rich lifestyle data also allowed us to assess potential effect modifications. We observed slightly stronger associations between the sulfur microbial diet score and CRC risk among participants who did not regularly use aspirin and those who ever smoked. Because regular aspirin use is an established CRC protective factor and smoking is an established CRC risk factor,^42,43^ these results suggest potential synergistic associations of diet, aspirin, and smoking with CRC risk.

Our study also has some limitations. Our participants were US health professionals, which constituted a relatively homogeneous population that minimizes sociodemographic confounding but may limit generalizability to other populations. Despite the careful adjustment for various risk factors in the multivariable model, we acknowledge that residual confounding and measurement error were possible in this observational study.

## Conclusions

This cohort study found that adherence to the sulfur microbial diet, characterized by high intakes of low-calorie drinks, red meats, and processed meats, and low intakes of fruits, whole grains, and vegetables, was associated with increased risk of CRC, suggesting a plausible microbial mediation for diet-CRC associations and the potential of using dietary modification as a strategy for risk reduction in CRC. Further epidemiologic and mechanistic studies are needed to delineate the biological pathways underlying the interplay of diet, other host risk factors, and the gut microbiome in CRC development.

## Notes

Supplement. **eFigure.** Sample Flow Diagram **eTable 1.** List of Sulfur-Metabolizing Bacteria **eTable 2.** Age-Adjusted Baseline Characteristics (2013) of the Men’s Lifestyle Validation Study (MLVS) and Mind Body Study (MBS) Participants **eTable 3.** Representative Food Items for Food Groups Included in the Sulfur Microbial Diet Score **eTable 4.** Age-Adjusted Baseline Dietary Intakes in Servings per Week Among Men [Health Professionals Follow-up Study (HPFS)] and Women [Nurses’ Health Study (NHS) and Nurses’ Health Study II (NHS2)] by Quintiles of Sulfur Microbial Diet Score **eTable 5.** Hazard Ratios (HR) and 95% Confidence Intervals (CI) of Colorectal Cancer Incidence in Men [Health Professionals Follow-up Study (HPFS)] and Women [Nurses’ Health Study (NHS) and Nurses’ Health Study II (NHS2)] by Quintiles of Sulfur Microbial Diet Score **eTable 6.** Multivariable-Adjusted Association Between the Sulfur Microbial Diet Score and Colorectal Cancer Incidence in the Pooled Analytic Cohort of Health Professionals Follow-up Study (HPFS), Nurses’ Health Study (NHS), and Nurses’ Health Study II (NHS2), With Additional Adjustment for the Western Dietary Pattern Score

## References

[1] Sung H, Ferlay J, Siegel RL, . Global cancer statistics 2020: GLOBOCAN estimates of incidence and mortality worldwide for 36 cancers in 185 countries. CA Cancer J Clin. 2021;71(3):209-249. doi:10.3322/caac.21660 33538338

[2] Magee EA, Richardson CJ, Hughes R, Cummings JH. Contribution of dietary protein to sulfide production in the large intestine: an in vitro and a controlled feeding study in humans. Am J Clin Nutr. 2000;72(6):1488-1494. doi:10.1093/ajcn/72.6.1488 11101476

[3] Ijssennagger N, van der Meer R, van Mil SWC. Sulfide as a mucus barrier-breaker in inflammatory bowel disease? Trends Mol Med. 2016;22(3):190-199. doi:10.1016/j.molmed.2016.01.002 26852376

[4] Attene-Ramos MS, Wagner ED, Gaskins HR, Plewa MJ. Hydrogen sulfide induces direct radical-associated DNA damage. Mol Cancer Res. 2007;5(5):455-459. doi:10.1158/1541-7786.MCR-06-0439 17475672

[5] David LA, Maurice CF, Carmody RN, . Diet rapidly and reproducibly alters the human gut microbiome. Nature. 2014;505(7484):559-563. doi:10.1038/nature12820 24336217 PMC3957428

[6] Nguyen LH, Ma W, Wang DD, . Association between sulfur-metabolizing bacterial communities in stool and risk of distal colorectal cancer in men. Gastroenterology. 2020;158(5):1313-1325. doi:10.1053/j.gastro.2019.12.029 31972239 PMC7384232

[7] Palmisano BT, Zhu L, Eckel RH, Stafford JM. Sex differences in lipid and lipoprotein metabolism. Mol Metab. 2018;15:45-55. doi:10.1016/j.molmet.2018.05.008 29858147 PMC6066747

[8] Kim SE, Paik HY, Yoon H, Lee JE, Kim N, Sung MK. Sex- and gender-specific disparities in colorectal cancer risk. World J Gastroenterol. 2015;21(17):5167-5175. doi:10.3748/wjg.v21.i17.5167 25954090 PMC4419057

[9] Lieberman DA, Williams JL, Holub JL, . Race, ethnicity, and sex affect risk for polyps >9 mm in average-risk individuals. Gastroenterology. 2014;147(2):351-358. doi:10.1053/j.gastro.2014.04.037 24786894 PMC4121117

[10] Benedix F, Kube R, Meyer F, Schmidt U, Gastinger I, Lippert H; Colon/Rectum Carcinomas (Primary Tumor) Study Group. Comparison of 17,641 patients with right- and left-sided colon cancer: differences in epidemiology, perioperative course, histology, and survival. Dis Colon Rectum. 2010;53(1):57-64. doi:10.1007/DCR.0b013e3181c703a4 20010352

[11] Abancens M, Bustos V, Harvey H, McBryan J, Harvey BJ. Sexual dimorphism in colon cancer. Front Oncol. 2020;10:607909. doi:10.3389/fonc.2020.607909 33363037 PMC7759153

[12] Baran B, Mert Ozupek N, Yerli Tetik N, Acar E, Bekcioglu O, Baskin Y. Difference between left-sided and right-sided colorectal cancer: a focused review of literature. Gastroenterology Res. 2018;11(4):264-273. doi:10.14740/gr1062w 30116425 PMC6089587

[13] Zhang Z, Hyun JE, Thiesen A, . Sex-specific differences in the gut microbiome in response to dietary fiber supplementation in IL-10–deficient mice. Nutrients. 2020;12(7):2088. doi:10.3390/nu12072088 32679670 PMC7400915

[14] Daly CM, Saxena J, Singh J, . Sex differences in response to a high fat, high sucrose diet in both the gut microbiome and hypothalamic astrocytes and microglia. Nutr Neurosci. Published online April 16, 2020. doi:10.1080/1028415X.2020.1752996 32297553 PMC7572529

[15] Huang T, Trudel-Fitzgerald C, Poole EM, . The Mind-Body Study: study design and reproducibility and interrelationships of psychosocial factors in the Nurses’ Health Study II. Cancer Causes Control. 2019;30(7):779-790. doi:10.1007/s10552-019-01176-0 31049751 PMC6631300

[16] Witten DM, Tibshirani R, Hastie T. A penalized matrix decomposition, with applications to sparse principal components and canonical correlation analysis. Biostatistics. 2009;10(3):515-534. doi:10.1093/biostatistics/kxp008 19377034 PMC2697346

[17] Rimm EB, Giovannucci EL, Willett WC, . Prospective study of alcohol consumption and risk of coronary disease in men. Lancet. 1991;338(8765):464-468. doi:10.1016/0140-6736(91)90542-W 1678444

[18] Solomon CG, Willett WC, Carey VJ, . A prospective study of pregravid determinants of gestational diabetes mellitus. JAMA. 1997;278(13):1078-1083. doi:10.1001/jama.1997.03550130052036 9315766

[19] Bertoia ML, Rimm EB, Mukamal KJ, Hu FB, Willett WC, Cassidy A. Dietary flavonoid intake and weight maintenance: three prospective cohorts of 124,086 US men and women followed for up to 24 years. BMJ. 2016;352:i17. doi:10.1136/bmj.i17 26823518 PMC4730111

[20] Yuan C, Spiegelman D, Rimm EB, . Validity of a dietary questionnaire assessed by comparison with multiple weighed dietary records or 24-hour recalls. Am J Epidemiol. 2017;185(7):570-584. doi:10.1093/aje/kww104 28338828 PMC5859994

[21] Franzosa EA, Morgan XC, Segata N, . Relating the metatranscriptome and metagenome of the human gut. Proc Natl Acad Sci U S A. 2014;111(22):E2329-E2338. doi:10.1073/pnas.1319284111 24843156 PMC4050606

[22] Song SJ, Amir A, Metcalf JL, . Preservation methods differ in fecal microbiome stability, affecting suitability for field studies. mSystems. 2016;1(3):e00021-16. doi:10.1128/mSystems.00021-16 27822526 PMC5069758

[23] Voigt AY, Costea PI, Kultima JR, . Temporal and technical variability of human gut metagenomes. Genome Biol. 2015;16(1):73. doi:10.1186/s13059-015-0639-8 25888008 PMC4416267

[24] Mehta RS, Abu-Ali GS, Drew DA, . Stability of the human faecal microbiome in a cohort of adult men. Nat Microbiol. 2018;3(3):347-355. doi:10.1038/s41564-017-0096-0 29335554 PMC6016839

[25] McIver LJ, Abu-Ali G, Franzosa EA, . bioBakery: a meta’omic analysis environment. Bioinformatics. 2018;34(7):1235-1237. doi:10.1093/bioinformatics/btx754 29194469 PMC6030947

[26] Hu FB, Rimm E, Smith-Warner SA, . Reproducibility and validity of dietary patterns assessed with a food-frequency questionnaire. Am J Clin Nutr. 1999;69(2):243-249. doi:10.1093/ajcn/69.2.243 9989687

[27] Chen J, Bushman FD, Lewis JD, Wu GD, Li H. Structure-constrained sparse canonical correlation analysis with an application to microbiome data analysis. Biostatistics. 2013;14(2):244-258. doi:10.1093/biostatistics/kxs038 23074263 PMC3590923

[28] Kostic AD, Gevers D, Siljander H, ; DIABIMMUNE Study Group. The dynamics of the human infant gut microbiome in development and in progression toward type 1 diabetes. Cell Host Microbe. 2015;17(2):260-273. doi:10.1016/j.chom.2015.01.001 25662751 PMC4689191

[29] Mach N, Ruet A, Clark A, . Priming for welfare: gut microbiota is associated with equitation conditions and behavior in horse athletes. Sci Rep. 2020;10(1):8311. doi:10.1038/s41598-020-65444-9 32433513 PMC7239938

[30] Hu FB, Rimm EB, Stampfer MJ, Ascherio A, Spiegelman D, Willett WC. Prospective study of major dietary patterns and risk of coronary heart disease in men. Am J Clin Nutr. 2000;72(4):912-921. doi:10.1093/ajcn/72.4.912 11010931

[31] Dostal Webster A, Staley C, Hamilton MJ, . Influence of short-term changes in dietary sulfur on the relative abundances of intestinal sulfate-reducing bacteria. Gut Microbes. 2019;10(4):447-457. doi:10.1080/19490976.2018.1559682 30810441 PMC6748593

[32] Jiang J, Chan A, Ali S, . Hydrogen sulfide—mechanisms of toxicity and development of an antidote. Sci Rep. 2016;6:20831. doi:10.1038/srep20831 26877209 PMC4753484

[33] Kesse E, Clavel-Chapelon F, Boutron-Ruault M-C. Dietary patterns and risk of colorectal tumors: a cohort of French women of the National Education System (E3N). Am J Epidemiol. 2006;164(11):1085-1093. doi:10.1093/aje/kwj324 16990408 PMC2175071

[34] Dermadi D, Valo S, Ollila S, . Western diet deregulates bile acid homeostasis, cell proliferation, and tumorigenesis in colon. Cancer Res. 2017;77(12):3352-3363. doi:10.1158/0008-5472.CAN-16-2860 28416481

[35] Zoltick ES, Smith-Warner SA, Yuan C, . Sugar-sweetened beverage, artificially sweetened beverage and sugar intake and colorectal cancer survival. Br J Cancer. 2021;125(7):1016-1024. doi:10.1038/s41416-021-01487-7 34267328 PMC8476625

[36] Guercio BJ, Zhang S, Niedzwiecki D, . Associations of artificially sweetened beverage intake with disease recurrence and mortality in stage III colon cancer: results from CALGB 89803 (Alliance). PLoS One. 2018;13(7):e0199244. doi:10.1371/journal.pone.0199244 30024889 PMC6053135

[37] Wu QJ, Yang Y, Vogtmann E, . Cruciferous vegetables intake and the risk of colorectal cancer: a meta-analysis of observational studies. Ann Oncol. 2013;24(4):1079-1087. doi:10.1093/annonc/mds601 23211939 PMC3603442

[38] Song M, Garrett WS, Chan AT. Nutrients, foods, and colorectal cancer prevention. Gastroenterology. 2015;148(6):1244-60.e16. doi:10.1053/j.gastro.2014.12.035 25575572 PMC4409470

[39] Hashem FA, Motawea H, El-Shabrawy AE, Shaker K, El-Sherbini S. Myrosinase hydrolysates of *Brassica oleraceae* L. var. *italica* reduce the risk of colon cancer. Phytother Res. 2012;26(5):743-747. doi:10.1002/ptr.3591 22076869

[40] Bianchini F, Vainio H. Isothiocyanates in cancer prevention. Drug Metab Rev. 2004;36(3-4):655-667. doi:10.1081/DMR-200033468 15554241

[41] London SJ, Yuan J-M, Chung F-L, . Isothiocyanates, glutathione S-transferase M1 and T1 polymorphisms, and lung-cancer risk: a prospective study of men in Shanghai, China. Lancet. 2000;356(9231):724-729. doi:10.1016/S0140-6736(00)02631-3 11085692

[42] Freedman AN, Slattery ML, Ballard-Barbash R, . Colorectal cancer risk prediction tool for white men and women without known susceptibility. J Clin Oncol. 2009;27(5):686-693. doi:10.1200/JCO.2008.17.4797 19114701 PMC2645090

[43] Din FV, Theodoratou E, Farrington SM, . Effect of aspirin and NSAIDs on risk and survival from colorectal cancer. Gut. 2010;59(12):1670-1679. doi:10.1136/gut.2009.203000 20844293

