# Supplementary material for: Association Between the Sulfur Microbial Diet and Risk of Colorectal Cancer
Source: JAMA Netw Open. 2021 Nov 12;4(11):e2134308. doi: 10.1001/jamanetworkopen.2021.34308 (PMC8590167; doi:10.1001/jamanetworkopen.2021.34308)
Supplement: Supplement. — eFigure. Sample Flow Diagram eTable 1. List of Sulfur-Metabolizing Bacteria eTable 2. Age-Adjusted Baseline Characteristics (2013) of the Men’s Lifestyle Validation Study (MLVS) and Mind Body Study (MBS) Participants eTable 3. Representative Food Items for Food Groups Included in the Sulfur Microbial Diet Score eTable 4. Age-Adjusted Baseline Dietary Intakes in Servings per Week Among Men [Health Professionals Follow-up Study (HPFS)] and Women [Nurses’ Health Study (NHS) and Nurses’ Health Study II (NHS2)] by Quintiles of Sulfur Microbial Diet Score eTable 5. Hazard Ratios (HR) and 95% Confidence Intervals (CI) of Colorectal Cancer Incidence in Men [Health Professionals Follow-up Study (HPFS)] and Women [Nurses’ Health Study (NHS) and Nurses’ Health Study II (NHS2)] by Quintiles of Sulfur Microbial Diet Score eTable 6. Multivariable-Adjusted Association Between the Sulfur Microbial Diet Score and Colorectal Cancer Incidence in the Pooled Analytic Cohort of Health Professionals Follow-up Study (HPFS), Nurses’ Health Study (NHS), and Nurses’ Health Study II (NHS2), With Additional Adjustment for the Western Dietary Pattern Score [file jamanetwopen-e2134308-s001.pdf]

Supplementary Online Content

Wang Y, Nguyen LH, Mehta RS, Song M, Huttenhower C, Chan AT. Association between the sulfur microbial diet and risk of colorectal cancer. *JAMA Netw Open.* 2021;4(11):e2134308. doi:10.1001/jamanetworkopen.2021.34308

eFigure. Sample Flow Diagram
eTable 1. List of Sulfur-Metabolizing Bacteria
eTable 2. Age-Adjusted Baseline Characteristics (2013) of the Men's Lifestyle Validation Study (MLVS) and Mind Body Study (MBS) Participants
eTable 3. Representative Food Items for Food Groups Included in the Sulfur Microbial Diet Score
eTable 4. Age-Adjusted Baseline Dietary Intakes in Servings per Week Among Men [Health Professionals Follow-up Study (HPFS)] and Women [Nurses' Health Study (NHS) and Nurses' Health Study II (NHS2)] by Quintiles of Sulfur Microbial Diet Score
eTable 5. Hazard Ratios (HR) and 95% Confidence Intervals (CI) of Colorectal Cancer Incidence in Men [Health Professionals Follow-up Study (HPFS)] and Women [Nurses' Health Study (NHS) and Nurses' Health Study II (NHS2)] by Quintiles of Sulfur Microbial Diet Score
eTable 6. Multivariable-Adjusted Association Between the Sulfur Microbial Diet Score and Colorectal Cancer Incidence in the Pooled Analytic Cohort of Health Professionals Follow-up Study (HPFS), Nurses' Health Study (NHS), and Nurses' Health Study II (NHS2), With Additional Adjustment for the Western Dietary Pattern Score

This supplementary material has been provided by the authors to give readers additional information about their work.

© 2021 Wang Y et al. *JAMA Network Open.*

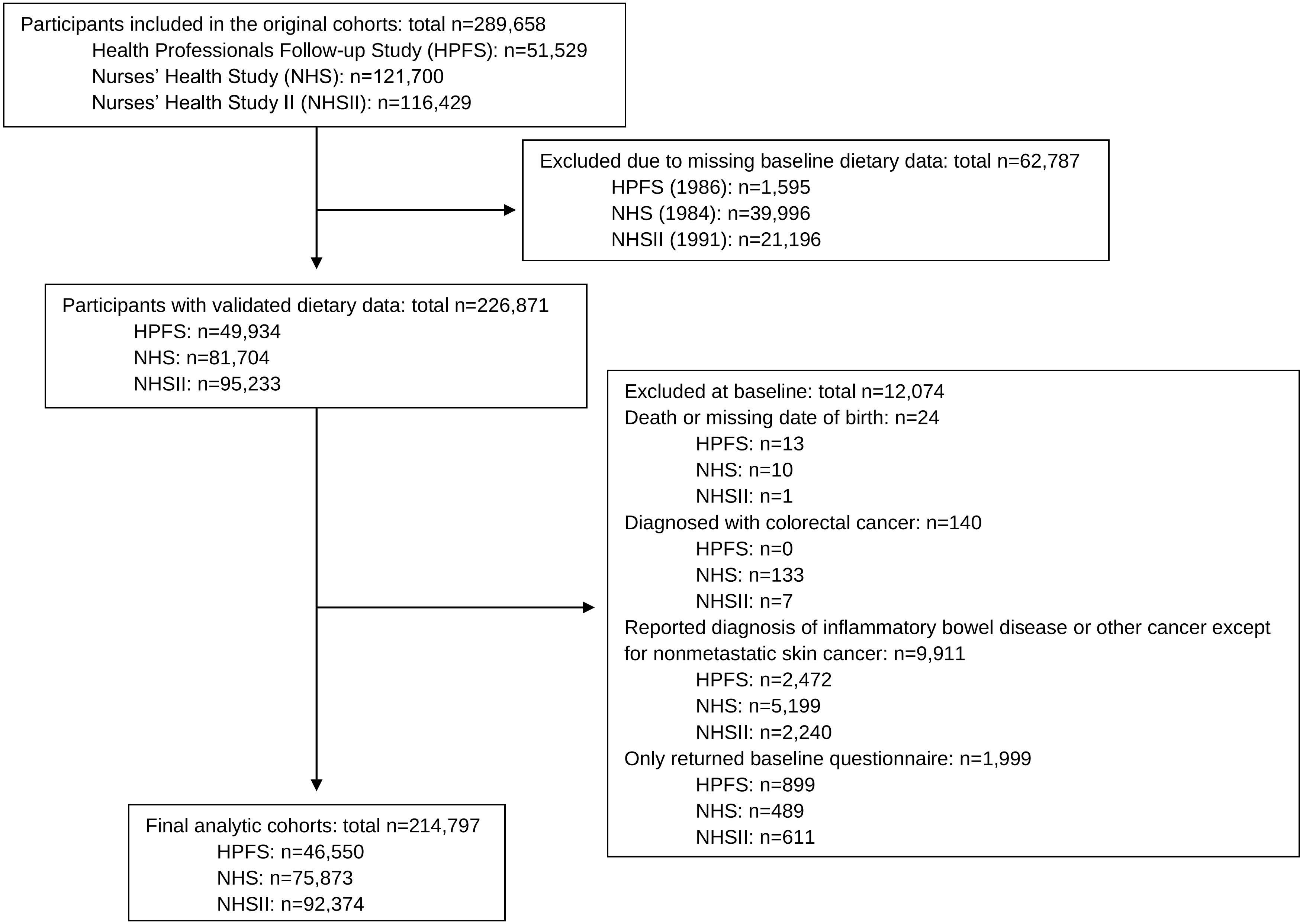

eFigure. Sample Flow Diagram

© 2021 Wang Y et al. *JAMA Network Open.*

**eTable 1.** List of Sulfur-Metabolizing Bacteria
Acidaminococcus unclassified
Adlercreutzia equolifaciens
Alistipes finegoldii
Alistipes putredinis
Anaerotruncus colihominis
Bacteroides clarus
Bacteroides intestinalis
Bacteroides ovatus
Bacteroides plebeius
Bacteroides stercoris
Bacteroides uniformis
Bacteroides vulgatus
Bilophila unclassified
Bilophila wadsworthia
Burkholderiales bacterium 1 1 47
Clostridiales bacterium 1 7 47FAA
Coprococcus catus
Desulfovibrio desulfuricans
Eggerthella lenta
Erysipelotrichaceae bacterium 21 3
Erysipelotrichaceae bacterium 6 1 45
Eubacterium ramulus
Eubacterium rectale
Gordonibacter pamelaeae
Lachnospiraceae bacterium 5 1 63FAA
Odoribacter splanchnicus
Oxalobacter formigenes
Parabacteroides distasonis
Parabacteroides goldsteinii
Parabacteroides johnsonii
Parabacteroides merdae
Parabacteroides unclassified
Paraprevotella clara
Paraprevotella xylaniphila
Parasutterella excrementihominis
Roseburia intestinalis
Ruminococcus bromii
Streptococcus australis
Streptococcus vestibularis
Sutterella wadsworthensis
Veillonella atypica
Veillonella parvula
Veillonella unclassified

© 2021 Wang Y et al. *JAMA Network Open*.

eTable 2. Age-Adjusted Baseline Characteristics (2013) of the Men's Lifestyle Validation Study (MLVS) and Mind Body Study (MBS) Participants, Mean (SD) or %

	MLVS	MBS
Age, years[a]	70.5 (4.3)	61.0 (3.8)
White race, %	94.5	95.8
BMI (kg/m²)	25.5 (3.7)	25.6 (5.3)
Physical activity, MET-hours/week	48.2 (36.2)	25.7 (18.7)
Current smoker, %	1.0	2.3
Antibiotic use in past year, %	18.6	25.4
Bowel preparation in past 2 months, %	5.2	1.9
Probiotic use in past 2 months, %	8.5	16.4
Total calorie intake, kcal/day	2169.4 (698.1)	1398.8 (467.4)
Bristol stool scale		
Hard stool (Type 1-2), %	5.2	14.6
Normal stool (Type 3-5), %	87.3	69.0
Loose stool (Type 6-7), %	7.5	14.1
Alcohol intake		
Never, %	13.5	15.5
Rarely, %	13.2	31.5
1-6 times per week, %	39.6	39.4
Daily, %	30.7	11.7
More than daily, %	3.0	1.9
Menopausal status		
Had period, %	-	0.5
No period, %	-	97.2
Period induced by hormone, %	-	1.9

Abbreviations: BMI, body mass index; MET, metabolic equivalent task.

Values are mean (standard deviation) unless noted as %.

[a] Not age-adjusted.

© 2021 Wang Y et al. *JAMA Network Open.*

**eTable 3.** Representative Food Items for Food Groups Included in the Sulfur Microbial Diet Score	
**Food groups**	**Representative food items**
Low-calorie beverages	Low-energy cola, other low-energy carbonated beverage
French fries	French fries
Red meats	Beef, pork, lamb, hamburger
Processed meats	Processed meats, bacon, hot dogs
Liquor	Liquor
Butter	Butter
High-fat dairy products	Whole milk, cream, sour cream, ice cream, cream cheese, other cheese
Coffee	Coffee
Sugar-sweetened beverages	Cola with sugar, other carbonated beverages with sugar, fruit drinks
Desserts	Chocolate bars or pieces, candy bars, cookies, brownies, doughnuts, cake, pie, sweet roll, coffee cake, pastry
Tomato	Tomatoes, tomato juice, tomato sauce
Fruit juice	Apple juice or cider, orange juice, grapefruit juice, other fruit juice
Poultry	Chicken or turkey with or without skin
Low-fat dairy products	Skim or low-fat milk, sherbet or ice milk, yogurt
Salad dressing	Oil and vinegar salad dressing
Beer	Beer
Nuts	Peanuts, other nuts, peanut butter
Fish	Canned tuna fish, dark-meat fish, other fish, shrimp, lobster, scallops
Other vegetables	Celery, mushrooms, green pepper, corn, mixed vegetables, eggplant, summer squash
Cruciferous vegetables	Broccoli; coleslaw and uncooked cabbage; cooked cabbage; cauliflower; Brussels sprouts; kale, mustard, and chard greens; sauerkraut
Leafy vegetables	Spinach, iceberg or head lettuce, romaine or leaf lettuce
Legumes	String beans, peas or lima beans, beans or lentils, tofu or soybeans, alfalfa sprouts
Whole grains	Cooked oatmeal, other cooked breakfast cereal, dark bread, brown rice, other grains, bran added to food, wheat germ
Yellow vegetables	Carrots, yellow (winter) squash, yams
Fruits	Raisins or grapes, avocado, bananas, cantaloupe, watermelon, fresh apples or pears, oranges, grapefruit, strawberries, blueberries, peaches, apricots, plums

© 2021 Wang Y et al. *JAMA Network Open.*

eTable 4. Age-Adjusted Baseline Dietary Intakes in Servings per Week Among Men (Health Professionals Follow-up Study [HPFS]) and Women (Nurses' Health Study [NHS] and Nurses' Health Study II [NHS2]) by Quintiles of Sulfur Microbial Diet Score[a]

	**Men (HPFS)**					**Women (NHS and NHSII)**				
	Q1	Q2	Q3	Q4	Q5	Q1	Q2	Q3	Q4	Q5
Low-calorie beverages	2.2 (4.3)	2.4 (4.4)	2.8 (4.7)	3.3 (5.4)	6.6 (10.6)	4.3 (6.8)	4.5 (6.7)	4.9 (7.3)	5.6 (8.1)	10.1 (12.9)
French fries	0.3 (0.6)	0.5 (0.7)	0.6 (0.8)	0.8 (0.9)	1.4 (1.5)	0.3 (0.5)	0.4 (0.5)	0.5 (0.6)	0.6 (0.7)	1.1 (1.2)
Red meats	2.9 (2.6)	3.6 (2.7)	4.1 (2.7)	4.7 (2.9)	6.3 (4)	4.1 (3.2)	4.8 (3.2)	5.2 (3.3)	5.6 (3.5)	7.2 (4.4)
Processed meats	1.4 (2)	2 (2.3)	2.5 (2.4)	2.9 (2.7)	4.3 (4.4)	1.2 (1.5)	1.5 (1.7)	1.8 (1.8)	2 (2)	2.8 (3)
Liquor	1.2 (3.1)	1.8 (4)	2.2 (4.4)	2.9 (5.5)	4.5 (7.8)	0.4 (1.5)	0.6 (2)	0.7 (2.2)	0.9 (2.8)	1.5 (4.4)
Butter	1.2 (3.3)	1.6 (3.8)	1.8 (4)	2.2 (4.7)	3.2 (6.3)	1.4 (3.5)	1.5 (3.7)	1.7 (4)	1.9 (4.4)	2.7 (5.7)
High-fat dairy products	5.4 (6)	6.2 (6.4)	6.7 (6.6)	7.2 (7.4)	8.5 (9)	6.4 (6.1)	6.7 (6.2)	6.8 (6.5)	7 (7)	7.8 (8.2)
Coffee	24.2 (20.7)	27.7 (20.7)	29.5 (20)	31.4 (19.9)	33.7 (20.1)	13 (12.2)	13.3 (12.2)	13.6 (12.4)	13.9 (12.7)	14.1 (13.7)
Sugar-sweetened beverages	1.7 (3.3)	2.1 (3.5)	2.3 (3.6)	2.8 (4.4)	3.3 (5.4)	1.9 (3.9)	2.3 (4.3)	2.6 (4.8)	3.1 (5.5)	3.9 (6.8)
Desserts	6.4 (7.9)	7.3 (8.3)	7.4 (8.2)	7.3 (7.8)	7.9 (8.6)	6.5 (7)	7.1 (7.3)	7.2 (7.3)	7.3 (7.3)	7.9 (8)
Tomato	5.9 (4.9)	4.6 (3.4)	4 (2.8)	3.5 (2.8)	3.2 (2.8)	5.1 (3.9)	3.9 (2.9)	3.4 (2.6)	3 (2.4)	2.6 (2.3)
Fruit juice	7.7 (8)	6.3 (6.1)	5.4 (5.2)	4.7 (4.8)	3.9 (4.5)	16.5 (9.1)	10.7 (5.4)	8.2 (4.5)	6 (3.8)	4.2 (3.4)
Poultry	3.1 (2.5)	2.7 (2)	2.4 (1.8)	2.1 (1.6)	1.9 (1.6)	5.7 (4)	4.8 (3)	4.1 (2.8)	3.6 (2.5)	3.2 (2.5)

© 2021 Wang Y et al. *JAMA Network Open.*

Low-fat dairy products	9.7 (8.8)	8 (7.7)	6.8 (7)	5.7 (6.5)	4.5 (5.9)	11.9 (8.7)	9.7 (7.7)	8 (7.2)	6.4 (6.5)	4.7 (5.7)
Salad dressing	2.6 (3.7)	2 (2.6)	1.6 (2.2)	1.3 (1.8)	1 (1.6)	3.2 (4)	2.3 (2.8)	1.8 (2.4)	1.4 (1.9)	1.1 (1.7)
Beer	1.7 (3.9)	2 (4.5)	2.1 (4.7)	2.1 (4.6)	1.9 (4.3)	0.7 (2.4)	0.7 (2.5)	0.7 (2.6)	0.7 (2.3)	0.6 (1.9)
Nuts	4.8 (6.6)	3.8 (5)	3.2 (4.2)	2.8 (3.5)	2.6 (3.6)	1.9 (2.8)	1.4 (1.9)	1.2 (1.6)	1 (1.4)	0.8 (1.2)
Fish	4.2 (3.2)	3.2 (2.3)	2.7 (1.9)	2.2 (1.6)	1.9 (1.6)	3.1 (2.5)	2.3 (1.7)	1.9 (1.4)	1.6 (1.2)	1.3 (1.2)
Other vegetables	5.8 (4.4)	4.1 (2.9)	3.3 (2.3)	2.8 (2)	2.3 (1.8)	7.4 (5.3)	5 (3.2)	4 (2.6)	3.3 (2.3)	2.8 (2.1)
Cruciferous vegetables	4.2 (3.6)	2.7 (2.1)	2.1 (1.7)	1.7 (1.4)	1.4 (1.3)	5.3 (4.3)	3.4 (2.3)	2.7 (1.9)	2.2 (1.7)	1.8 (1.5)
Leafy vegetables	8.2 (6.4)	5.9 (3.8)	4.8 (3.2)	3.9 (2.8)	3.3 (2.7)	8.9 (6.6)	5.9 (3.9)	4.8 (3.3)	3.8 (2.8)	3 (2.5)
Legumes	5 (3.6)	3.4 (2.2)	2.8 (1.8)	2.4 (1.7)	2.1 (1.6)	4.3 (3.2)	2.9 (1.9)	2.5 (1.6)	2.1 (1.5)	1.7 (1.4)
Whole grains	18.6 (13.3)	11.9 (8.5)	8.8 (6.9)	6.5 (5.8)	4.5 (5)	14.3 (9.8)	9.5 (7)	6.8 (5.6)	4.8 (4.5)	3.2 (3.7)
Yellow vegetables	0.7 (1.1)	0.3 (0.4)	0.2 (0.3)	0.2 (0.3)	0.1 (0.2)	5.3 (4.5)	2.9 (2)	2 (1.5)	1.5 (1.2)	1 (1)
Fruits	20.5 (12.3)	13.1 (6.8)	9.9 (5.5)	7.5 (4.9)	5.4 (4.4)	16.5 (9.1)	10.7 (5.4)	8.2 (4.5)	6 (3.8)	4.2 (3.4)

[a] Food groups are ordered based on weights in the sulfur microbial diet score in descending order.

© 2021 Wang Y et al. *JAMA Network Open.*

eTable 5. Hazard Ratios (HR) and 95% Confidence Intervals (CI) of Colorectal Cancer Incidence in Men [Health Professionals Follow-up Study (HPFS)] and Women [Nurses' Health Study (NHS) and Nurses' Health Study II (NHS2)] by Quintiles of Sulfur Microbial Diet Score[a]

Model	Sulfur microbial diet score (quintile)					*P*-trend[b]
	Q1 (lowest)	Q2	Q3	Q4	Q5 (highest)	
**Men (HPFS)**						
Cases	232	247	220	216	222	
Person-years	206,635	209,173	204,043	200,375	191,366	
Age-adjusted[c]	1 [ref.]	1.14 (0.95-1.37)	1.13 (0.94-1.36)	1.24 (1.02-1.49)	1.60 (1.32-1.94)	<.001
Multivariable-adjusted[d]	1 [ref.]	1.11 (0.93-1.34)	1.09 (0.90-1.32)	1.16 (0.95-1.41)	1.45 (1.18-1.77)	.001
**Women (NHS and NSII)**						
Cases	418	444	422	420	374	
Person-years	877,275	882,514	868,469	843,772	794,426	
Age-adjusted[c]	1 [ref.]	1.12 (0.98-1.28)	1.15 (1.00-1.31)	1.24 (1.08-1.43)	1.33 (1.15-1.54)	<.001
Multivariable-adjusted[d]	1 [ref.]	1.09 (0.95-1.24)	1.09 (0.95-1.26)	1.16 (1.00-1.34)	1.18 (1.01-1.37)	.02

[a] The Cochran's Q test for heterogeneity between men and women yielded a *p*-value of 0.17 in age-adjusted model and multivariable-adjusted model.
[b] Trend test was performed using median value of each diet score quintile as a continuous variable.
[c] Models were stratified by age, questionnaire cycle, and cohort.
[d] Models were stratified by age, questionnaire cycle, cohort, and adjusted for the following covariates: race, body mass index, family history of colorectal cancer, physical activity, smoking status, smoking packyears, menopausal hormone use (women only), aspirin use, non-steroid anti-inflammatory drug use, prior endoscopy, recent physical examination, and total calorie intake.

© 2021 Wang Y et al. *JAMA Network Open.*

eTable 6. Multivariable-Adjusted Association Between the Sulfur Microbial Diet Score and Colorectal Cancer Incidence in the Pooled Analytic Cohort of Health Professionals Follow-up Study (HPFS), Nurses' Health Study (NHS), and Nurses' Health Study II (NHS2), With Additional Adjustment for the Western Dietary Pattern Score

	**Sulfur microbial diet score (quintile)**					***P*-trend**[a]
	Q1 (lowest)	Q2	Q3	Q4	Q5 (highest)	
Hazard ratio (95% confidence interval)[b]	1 [ref.]	1.08 (0.97-1.21)	1.07 (0.95-1.21)	1.13 (0.99-1.28)	1.21 (1.05-1.40)	.01

[a] Trend test was performed using median value of each diet score quintile as a continuous variable.
[b] Models were stratified by age, questionnaire cycle, cohort, and adjusted for the following covariates: race, body mass index, family history of colorectal cancer, physical activity, smoking status, smoking packyears, menopausal hormone use (women only), aspirin use, non-steroid anti-inflammatory drug use, prior endoscopy, recent physical examination, total calorie intake, and the Western dietary pattern score, which was derived using principal component analysis.

© 2021 Wang Y et al. *JAMA Network Open.*
